# Vertebral cross-sectional growth: A predictor of vertebral wedging in the immature skeleton

**DOI:** 10.1371/journal.pone.0190225

**Published:** 2017-12-27

**Authors:** Ervin Poorghasamians, Patricia C. Aggabao, Tishya A. L. Wren, Skorn Ponrartana, Vicente Gilsanz

**Affiliations:** 1 Department of Radiology, Children’s Hospital Los Angeles, Keck School of Medicine, University of Southern California, Los Angeles, California, United States of America; 2 Division of Orthopaedic Surgery, Children’s Hospital Los Angeles, Keck School of Medicine, University of Southern California, Los Angeles, California, United States of America; 3 Department of Pediatrics, Children’s Hospital Los Angeles, Keck School of Medicine, University of Southern California, Los Angeles, California, United States of America; Medical College of Wisconsin, UNITED STATES

## Abstract

The degree of vertebral wedging, a key structural characteristic of spinal curvatures, has recently been found to be negatively related to vertebral cross-sectional area (CSA). The purpose of this longitudinal study was to examine the relation between vertebral cross-sectional growth and vertebral wedging progression within the immature lumbar spine. Using magnetic resonance imaging (MRI), we analyzed the potential association between increases in lumbar vertebral CSA and changes in L5 vertebral wedging in 27 healthy adolescent girls (ages 9–13 years) twice within a two-year period. Vertebral CSA growth was negatively associated with changes in posteroanterior vertebral wedging (*r* = -0.61; *p* = 0.001). Multiple regression analysis showed that this relation was independent of gains in age, height, and weight. When compared to the 14 girls whose vertebral wedging progressed, the 13 subjects whose vertebral wedging decreased had significantly greater vertebral cross-sectional growth (0.39 ± 0.25 vs. 0.75 ± 0.23 cm^2^; *p* = 0.001); in contrast, there were no significant differences in increases in age, height, or weight between the two groups. Changes in posteroanterior vertebral wedging and the degree of lumbar lordosis (LL) positively correlated (*r* = 0.56, *p* = 0.002)—an association that persisted even after adjusting for gains in age, height, and weight. We concluded that in the immature skeleton, vertebral cross-sectional growth is an important determinant of the plasticity of the vertebral body; regression of L5 vertebral wedging is associated with greater lumbar vertebral cross-sectional growth, while progression is the consequence of lesser cross-sectional growth.

## Introduction

Just as the term plasticity in engineering exemplifies the ability of solid material to undergo deformation in response to load, skeletal plasticity describes the facility of growing bone to alter modelling as a consequence of mechanical stresses [1]. Contrary to the permanent deformations that adult vertebrae sustain under load, asymmetrical vertebral growth in children has the capacity to change shape in response to mechanical stresses [2,3].

Wedging of the vertebral body, a key structural characteristic of spinal curvatures [4,5,6,7], is the result of a simultaneous increase in longitudinal growth on the convex side of a curve and inhibition on the concave side [8,9,10]. We recently found a negative correlation between values for vertebral cross-sectional area (CSA) and both lateral vertebral wedging in scoliosis and posteroanterior vertebral wedging within the lumbar curve [4]—relations that were independent of age and body size. Since vertebral CSA is a major determinant of spinal flexibility and strength [11,12,13,14,15], asymmetrical vertebral growth secondary to unbalanced axial load would be most prominent in the presence of small vertebral cross-sectional dimensions.

In the current study, we examined the potential for wedge deformities in immature vertebrae to reshape or resolve over time. Using magnetic resonance imaging (MRI), we analyzed the relations between lumbar vertebral cross-sectional growth and changes in the degree of L5 vertebral wedging in healthy adolescent girls. We hypothesized that vertebral cross-sectional growth is a major determinant of vertebral body plasticity and negatively correlated to changes in vertebral wedging. We predicted that greater lumbar vertebral cross-sectional growth would result in diminished or even regression of L5 vertebral wedging, while lesser cross-sectional growth would lead to progressive wedging.

## Materials and methods

The study protocol was approved by the Institutional Review Board (IRB) for Clinical Investigations at Children’s Hospital Los Angeles (CHLA), which was compliant with the Declaration of Helsinki and the Health Insurance Portability and Accountability Act. Written assent and consent were obtained from all subjects and their parent(s). All study subjects were recruited from the Division of General Pediatrics at CHLA, and were included in a previous cross-sectional investigation [4]. For the purpose of this study, adolescent girls with at least three cm increase in height, who continued having normal physical examinations a year or more later, were eligible to participate. Using these criteria, a total of 27 healthy girls between the ages of 9–13 years underwent follow-up examinations of their lumbar spine one to two years later.

Baseline and follow-up MRI examinations were performed in the supine position with extended legs and without the use of general anesthesia or contrast enhancement using a 3.0 Tesla whole-body MRI scanner (Achieva R3.2, Philips Healthcare, Cleveland, Ohio) with a standard 15-channel spine coil. Three dimensional T2-weighted turbo spin echo scans were taken with TE of 120 ms, TR of 3000 ms, a flip angle of 90°, and with a voxel size of 1.0 x 1.0 x 1.0 mm. Vertebral CSA of the lumbar vertebral bodies, posteroanterior vertebral wedging of L5, and LL angle were measured as previously described [4,14]. Briefly, LL was measured in the sagittal plane as the angle between the superior endplate of L1 and the inferior endplate of L5. Vertebral CSA was measured in the axial plane at the midportion of the vertebral body based on the anterior and posterior heights of the lumbar vertebrae; an average of all 5 lumbar vertebrae was used. Posteroanterior vertebral wedging was defined as the angle between the superior and inferior endplates at the midsagittal plane of L5. The coefficient of variation for repeated MRI measurements of vertebral CSA, LL angle, and vertebral wedging are between 0.8–3.0% [4,14]. All measurements were analyzed offline manually with image processing software (Osirix; Pixmeo, Switzerland). The change in a variable is defined as the difference between the values obtained at the second and first time points, and is denoted by a delta (Δ).

The data were analyzed using paired and unpaired *t* tests, and simple and multiple linear regression analyses using Statview software (version 5.0.1; SAS Institute, Cary, NC). Statistical significance was considered a *P* < 0.05. All values are expressed as mean ± SD.

## Results

The age, height, weight, and MRI characteristics of spinal morphology of the 27 girls at baseline and 13 to 18 months later are described in Table 1. As expected, values for height, weight, and the CSA of the vertebral body were significantly greater at follow-up. In contrast, vertebral wedging and LL angles did not significantly change between studies.

**Table 1:** Age, anthropometric, and MRI measurements of lumbar spine morphology in 27 healthy girls at baseline and follow-up.

	Baseline			Follow-Up			Change			*p* Value
Age (*yr*)	11.2	±	1.30	12.4	±	1.27	1.22	±	0.09	<0.0001
Height (*cm*)	147.9	±	9.60	154.2	±	8.42	6.39	±	2.31	<0.0001
Weight (*kg*)	41.7	±	10.4	48.8	±	11.4	7.14	±	3.44	<0.0001
Lumbar Vertebral CSA (*cm*^*2*^)	7.96	±	1.04	8.52	±	1.07	0.56	±	0.30	<0.0001
Vertebral Wedging (*°*)	13.1	±	3.01	13.2	±	2.53	0.11	±	2.97	0.849
Lumbar Lordosis (*°*)	26.6	±	8.93	28.9	±	8.76	2.28	±	5.59	0.044

**Fig 1:**
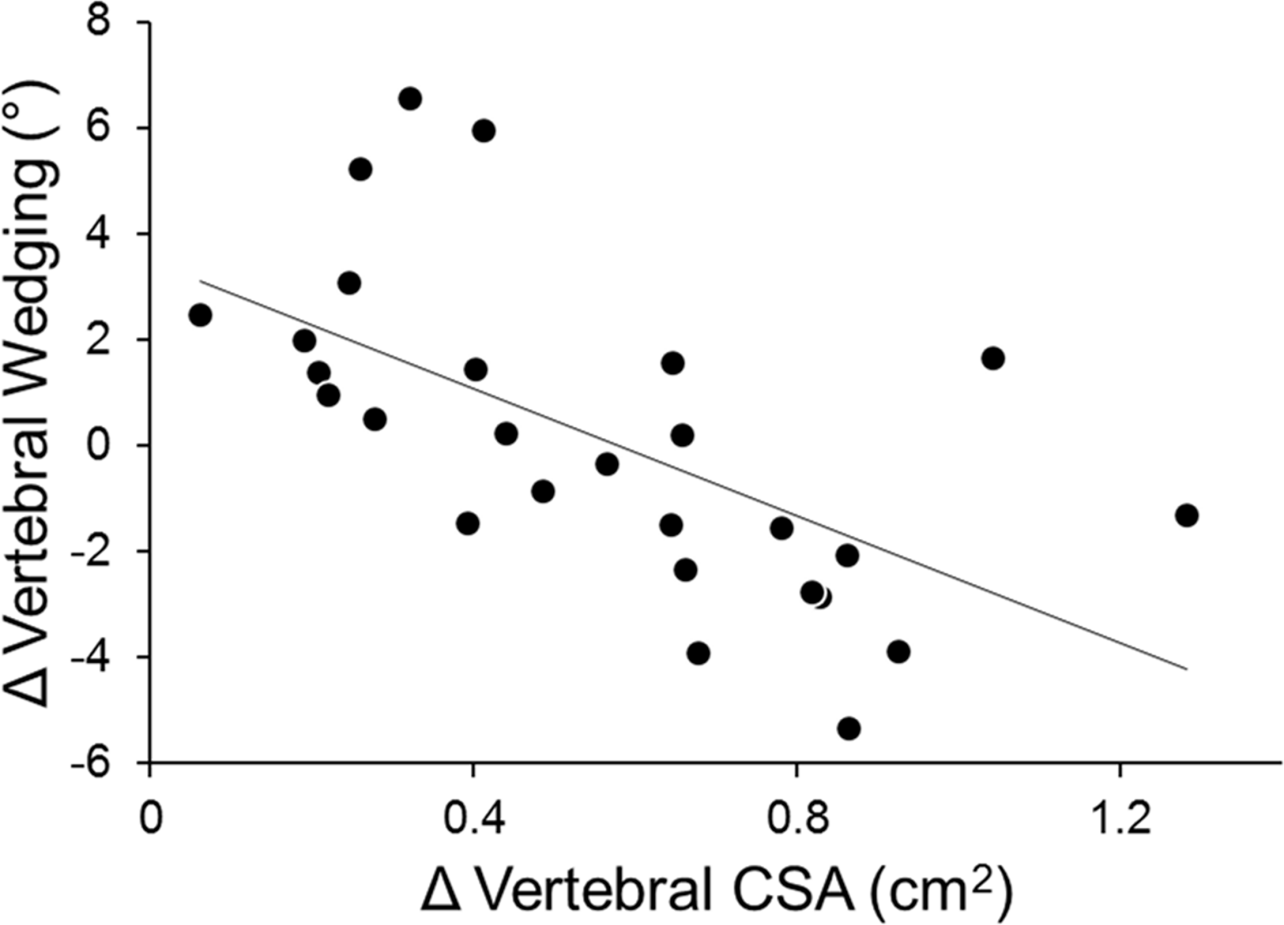
Simple linear regression between changes in vertebral CSA and vertebral wedging (*r* = -0.611; *p* = 0.001).

Changes in vertebral wedging angle did not correlate with increases in age or weight, but negatively correlated to increases in height (*r* = **-**0.49; *p* = 0.010). A negative correlation was also observed between increases in vertebral CSA and changes in vertebral wedging (*r* = -0.61; *p* = 0.001) (Fig 1). Overall, girls with the smallest vertebral cross-sectional growth had the greatest gains in vertebral wedging. Multiple linear regression analysis of the independent effects of increases in age, height, weight, and vertebral CSA on changes in vertebral wedging indicated that vertebral CSA growth was the sole variable that entered into the model (Table 2).

**Table 2:** Multiple linear regression model relating gains in age, height, weight, and vertebral CSA to changes in vertebral wedging.

	β	95% CI	*p* Value	R^2^
Δ *Vertebral Wedging (*°*)*				0.442
Δ Age (yr)	3.405	-10.107, 16.916	0.607	
Δ Height (cm)	-0.235	-0.752, 0.282	0.356	
Δ Weight (kg)	-0.174	-0.481, 0.133	0.252	
Δ Vertebral CSA (cm^2^)	-5.783	-10.320, -1.245	0.015	

**Fig 2:**
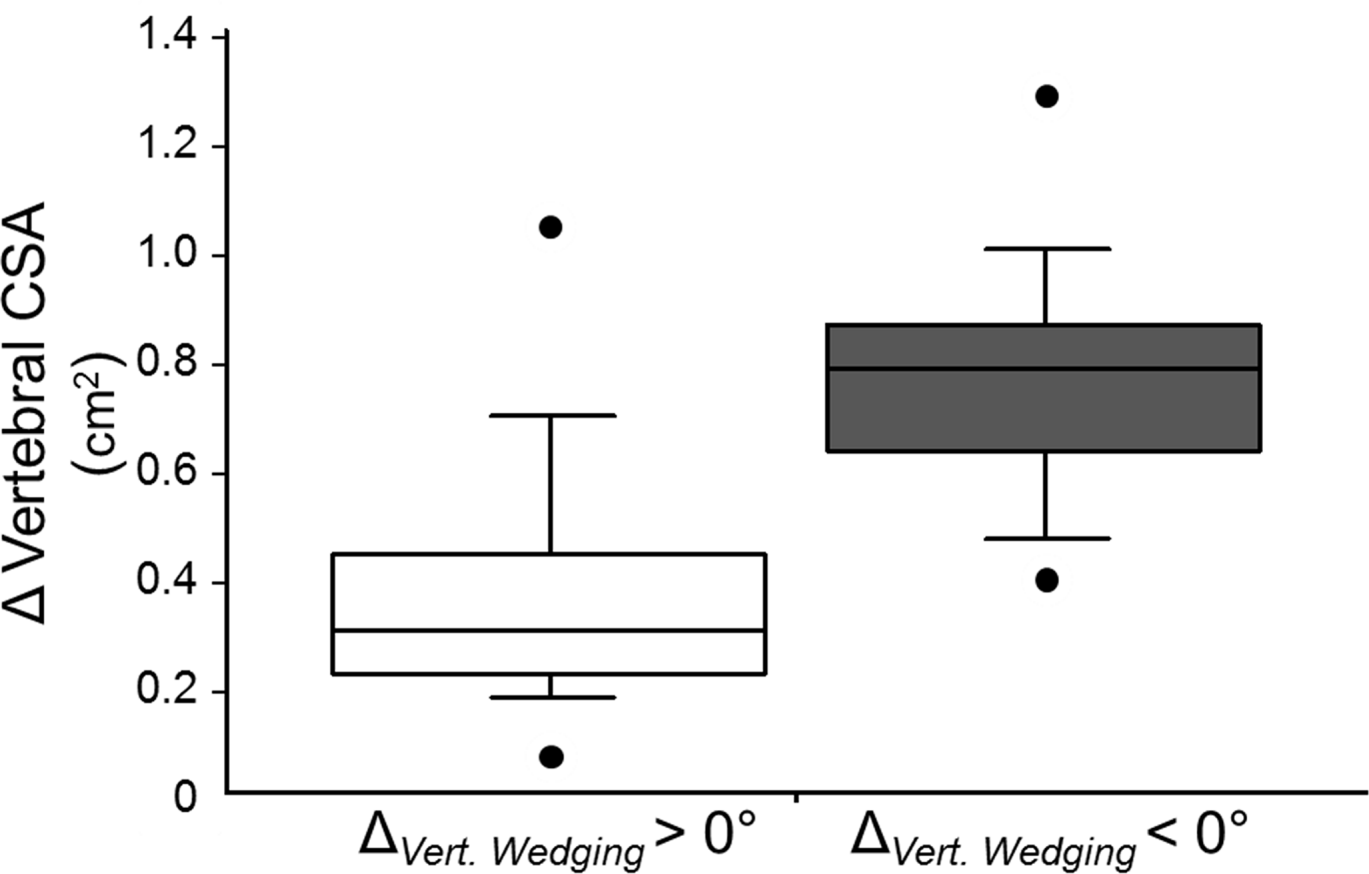
Boxplot showing differences in vertebral cross-sectional growth between girls with increased (*n* = 14) and decreased vertebral body wedging (*n* = 13); *p* = 0.001.

Although vertebral CSA increased in all subjects, vertebral cross-sectional growth was significantly lower in the 14 girls in which the vertebral wedging increased (Table 3). Notably, the 90th percentile for vertebral CSA gains in subjects with increased vertebral wedging corresponded to ~50th percentile in girls with decreased vertebral wedging (Fig 2). In contrast, there were no significant differences in increases in age, height, or weight between the two groups (Table 3).

**Table 3:** Changes in age, anthropometric measures, and lumbar spine morphology of girls with increased and deceased vertebral wedging.

	Increased Wedging			Decreased Wedging			*p* Value
	(n = 14)			(n = 13)			
Δ Age (yr)	1.2	±	0.07	1.3	±	0.10	0.091
Δ Height (cm)	5.6	±	2.10	7.3	±	2.26	0.049
Δ Weight (kg)	7.1	±	3.78	7.2	±	3.17	0.895
Δ Lumbar Vertebral CSA (cm^2^)	0.39	±	0.25	0.75	±	0.23	0.001
Δ Vertebral Wedging (°)	2.4	±	2.09	-2.3	±	1.41	<0.0001
Δ Lumbar Lordosis (°)	4.0	±	6.90	0.5	±	3.02	0.103

There were no significant correlations of height with the degree of LL either at baseline or follow-up (*p*’s > 0.05), whereas age correlated to degree of LL at both time points (*r*’s = 0.42 and 0.41; both *p*’s < 0.04). Weight, however, correlated to LL angle at baseline (*r* = 0.50; *p* = 0.008), but not at follow-up (*r* = 0.33; *p* = 0.098). Conversely, vertebral wedging positively correlated to LL at both time points (*r*’s = 0.57 and 0.60; both *p*’s < 0.002). Multiple regression analysis indicated that vertebral wedging was an independent predictor of LL both at baseline and follow-up (Table 4). Additionally, there was a positive correlation between changes in vertebral wedging and LL angles (*r* = 0.56, *p* = 0.002).When changes in LL were used as the dependent variable to examine their relationship to age, anthropometric measures, and vertebral wedging over time, the latter was the only significant independent variable in the model (Table 5).

**Table 4:** Multiple linear regressions of baseline and follow-up lordosis as a function of age, height, weight, and vertebral wedging.

Baseline	**β**	**95% CI**	***p* Value**	**R**^**2**^
*Lumbar Lordosis (°)*				0.664
Age (yr)	2.916	0.117, 5.715	0.042	
Height (cm)	-0.237	-0.730, 0.255	0.328	
Weight (kg)	0.394	0.029, 0.758	0.036	
Vertebral Wedging (°)	1.802	1.023, 2.582	<0.0001	
Follow-Up	**β**	**95% CI**	***p* Value**	**R**^**2**^
*Lumbar Lordosis (°)*				0.480
Age (yr)	2.301	-0.759, 5.361	0.133	
Height (cm)	-0.285	-0.870, 0.299	0.322	
Weight (kg)	0.212	-0.164, 0.589	0.255	
Vertebral Wedging (°)	1.857	0.726, 2.988	0.003	

**Table 5:** Multiple linear regression model on the effect of changes in vertebral wedging, after adjusting for gains in age, height, and weight, on lumbar lordosis progression.

	β	95% CI	*p* Value	R^2^
*Δ Lumbar Lordosis(°)*				0.361
Δ Age (yr)	0.606	-23.595, 24.807	0.959	
Δ Height (cm)	0.593	-0.415, 1.601	0.235	
Δ Weight (kg)	-0.034	-0.624, 0.555	0.906	
Δ Vertebral Wedging (°)	1.283	0.506, 2.060	0.002	

## Discussion

We recently found that smaller vertebral CSA is associated with greater vertebral wedging in scoliosis and lumbar lordosis curves [4]. In the current longitudinal study, we provide further evidence that vertebral cross-sectional growth is a major determinant of variations in vertebral wedging within the immature lumbar spine. Although vertebral CSA increased in all girls, those with the smallest increases in lumbar vertebral CSA during the adolescent growth spurt had the greatest progression in vertebral wedging—an association that persisted even after accounting for changes in age, height, and weight. When compared to healthy adolescent girls whose vertebral wedging progressed, subjects whose vertebral wedging decreased had on average 90% greater vertebral cross-sectional growth. These findings underscore the importance of vertebral cross-sectional growth as a modulator of vertebral wedging progression and highlight the plasticity of the immature spine in reshaping vertebral wedge deformities.

Accumulating evidence suggests that vertebral CSA is a structural determinant of spinal strength and flexibility [4,11,12,13,14,15]. During axial compressive loading, stress within the vertebral body is directly proportional to the applied force and inversely proportional to its cross-sectional dimensions [13,16]; a small vertebral CSA therefore imparts a mechanical disadvantage that increases stress within the vertebrae for all physical activities [17,18]. Since vertebral CSA is also inversely related to spinal flexibility, small cross-sectional dimensions would increase the magnitude of asymmetric loading on the vertebrae due to a larger range of motion. In the presence of disproportional loading, like that in LL, longitudinal bone growth is inhibited on the concave side of a curvature while accelerated on the convex side [3,7,19,20,21]. Hence, the two biomechanical properties associated with small vertebral cross-sectional growth, lesser strength and greater spinal flexibility, concurrently present a possible mechanism for the development and progression of vertebral wedging. As both properties are likely determinants of vertebral wedging, yet markedly different, it becomes very difficult to assess causality and establish which comes first [22].

Likewise, it is also challenging to determine whether increased wedging leads to greater lumbar lordosis or vice versa. The results of the current study are, nevertheless, consistent with prior investigations reporting a strong association between lumbar vertebral wedging and LL [4,7]. We found the degree of posteroanterior L5 wedging to be positively correlated to LL angle at both baseline and follow-up examinations—relations that persisted even after accounting for age, height, and weight. Moreover, multiple regression analysis indicated that LL progression was primarily predicted by gains in vertebral wedging.

This study is not the first to suggest that the plasticity of the immature vertebral body includes the potential for correcting asymmetric growth in response to changes in mechanical stress. Makino and colleagues recently investigated the plasticity of vertebral wedge deformities in the immature skeleton of adolescents with idiopathic scoliosis (AIS). They showed the degree of wedging in thoracic vertebrae to diminish a year after posterior corrective surgery [19]. A similar phenomenon occurs in pediatric patients with vertebral fractures secondary to leukemia or hypercorticolism [23,24,25,26,27,28,29]. In contrast to the mature skeleton, these pathological fractures frequently regain normal dimensions following treatment [29,30,31,32]. Interestingly, older teenagers, like adults, are less likely to regain vertebral body height [31,33,34], supporting the notion that vertebral body plasticity is a property of the immature axial skeleton.

There are several limitations in this study. Notably, we confined our investigation to the analysis of LL in adolescent girls, and whether our findings are generalizable to males, older populations, or other spinal curvatures is unknown. We chose to examine growing females since anterior lumbar curves increase markedly during adolescence [35], and are more prominent in females than in males [36,37,38,39,40]. Additionally, females have significantly smaller vertebral cross-sectional dimensions than males–a difference that is independent of body size and present throughout life [13,41,42,43]. The smaller female vertebral CSA is associated with greater spinal flexibility and likely facilitates the increased LL needed during pregnancy [44]. We selected girls due to their smaller vertebrae, greater degree of LL, and higher risk for vertebral wedging. While progressive scoliosis is most prevalent in adolescent females and vertebral wedging is also closely related to scoliosis severity [19,20,21], lateral spinal curvatures are complex deformities with multi-dimensional wedging progression [8]. By examining LL, we restricted the analysis of vertebral deformation to a single plane reducing the complexity and inaccuracies of measurements.

This study also has other methodological limitations. Since spinal morphology was determined using MRI, measures for LL were confined to the supine orientation. Prior reports, however, have shown that measures of LL in the upright and supine positions can be interchangeable when the lower extremities are straightened [45]. We also acknowledge that vertebral wedging was only measured in a single lumbar vertebra (L5); yet, among all the vertebral bodies of the lumbar spine, L5 has the greatest degree of posteroanterior wedging, and is the greatest contributor to the degree of lumbar curvature [4,35,46]. Lastly, our study was limited to a relatively short follow-up. However, all participants had at least a 3-centimeter increase in height, and prior data shows the degree of tracking for measures of vertebral CSA to be high and comparable to that of height throughout development [47]. Knowledge that children with small vertebral CSA will likely continue to have low values as young adults, highlights our potential ability to identify those children at risk to develop progressive wedging.

In conclusion, the current study provides evidence that vertebral cross-sectional growth is a key determinant of vertebral body plasticity in the immature lumbar spine. Vertebral wedging regressed in girls with the greatest vertebral cross-sectional growth, while those with the least growth experienced progression of the wedge deformity and LL. A better understanding of the reshaping potential of the growing vertebral body could aid in the design of novel preventative and corrective treatments for wedge deformities in children.

## Supporting information

S1 Table Ages, anthropometric characteristics, and MRI measures of vertebral morphology and degree of lumbar lordosis included in the analyses. (XLSX)
